# A Preoperative Risk-Prediction Model (SSPAL) for Prolonged Air Leak After Lobectomy for Lung Cancer: Development and Internal Validation

**DOI:** 10.3390/jcm15218367

**Published:** 2026-10-27

**Authors:** Yunus Türk, Necati Çitak, Esra Yamansavcı Şirzai, Ahmet Üçvet

**Affiliations:** Department of Thoracic Surgery, Dr. Suat Seren Chest Diseases and Surgery Training and Research Hospital, Izmir 35110, Turkey; necomomus@gmail.com (N.Ç.); yamansavci@gmail.com (E.Y.Ş.); ahmetucvet@hotmail.com (A.Ü.)

**Keywords:** carcinoma, non-small-cell lung, lobectomy, air leak, risk assessment, logistic models, predictive value of tests

## Abstract

**Background:** Prolonged air leak (PAL) remains one of the most common complications following lobectomy for non-small-cell lung cancer (NSCLC), leading to prolonged hospitalization, increased healthcare costs, and delayed recovery. We aimed to develop and internally validate a practical model for predicting PAL after lobectomy. **Methods:** This retrospective single-center cohort study included 905 consecutive patients who underwent lobectomy for NSCLC between 2017 and 2024. Patients were randomly allocated to derivation (*n* = 680) and internal validation (*n* = 225) cohorts (3:1). Candidate clinical, operative, and pathological variables were evaluated to identify predictors of PAL, and multivariable logistic regression was used to develop the Suat Seren Prolonged Air Leak (SSPAL) model. Model performance was assessed by discrimination (AUC) and calibration. **Results:** The overall incidence of PAL was 20.8%. Multivariable logistic regression identified lower body mass index (OR, 0.915; 95% CI, 0.871–0.961; *p* < 0.001), current smoking (OR, 4.096; 95% CI, 1.359–12.350; *p* = 0.01), and left-sided lobectomy (OR, 0.637; 95% CI, 0.424–0.956; *p* = 0.03) as independent predictors of PAL. Sex (*p* = 0.07) and upper lobectomy (*p* = 0.08) were retained in the final model. The SSPAL model demonstrated acceptable discrimination in the derivation cohort (AUC, 0.723; 95% CI, 0.675–0.771) and the internal validation cohort (AUC, 0.728; 95% CI, 0.649–0.807). **Conclusions:** The SSPAL model is a simple candidate risk-prediction model for estimating PAL risk after lobectomy for NSCLC. Prospective multicenter external validation is required before its clinical utility and potential role in decision-making can be established.

## 1. Introduction

Lung cancer remains the leading cause of cancer-related mortality worldwide despite advances in early diagnosis and multimodal treatment [1]. For patients with resectable NSCLC, surgical resection remains the cornerstone of curative treatment [2]. However, postoperative complications continue to adversely affect patient outcomes and healthcare resource utilization [3,4,5].

Among postoperative complications, prolonged air leak (PAL) is one of the most common after lobectomy, with reported incidences of 7–20% [3,6,7,8,9,10,11]. PAL is associated with prolonged chest tube duration, postoperative pain, pulmonary complications, increased healthcare costs, and delayed initiation of adjuvant therapy [3,5,7,8,9,10].

Over the past two decades, several prediction models have been developed to identify patients at increased risk of PAL [3,4,7,8,9,12,13,14]. Previous studies identified impaired pulmonary function, upper lobectomy, and pleural adhesions as major predictors of PAL [7,8]. Subsequently, large registry-based models, including the European Society of Thoracic Surgeons (ESTS) score developed by Pompili et al., incorporated demographic and operative variables including BMI, FEV_1_, sex, and type of pulmonary resection [12].

Despite these advances, the clinical applicability of existing prediction models remains limited. Most available models were developed using heterogeneous surgical populations that included different resection procedures, such as wedge resection, segmentectomy, bilobectomy, and lobectomy, each with distinct postoperative air leak profiles. In addition, variability in PAL definitions, model complexity, and limited validation have hindered broader clinical implementation [3,4,9,12,15]. Consequently, there remains a need for a simple, reliable, and clinically applicable prediction model derived from a homogeneous cohort of patients undergoing lobectomy and capable of providing risk estimates before surgery. We aimed to identify predictors of PAL after lobectomy for NSCLC and develop and internally validate the Suat Seren Prolonged Air Leak (SSPAL) prediction model.

## 2. Materials and Methods

### 2.1. Study Design and Patient Selection

This retrospective single-center cohort study included consecutive adults who underwent lobectomy for NSCLC between 1 January 2017, and 31 December 2024, at Suat Seren Chest Diseases and Thoracic Surgery Training and Research Hospital, Türkiye. The study was approved by the Institutional Non-Interventional Clinical Research Ethics Committee (Approval No. 2025/36-30, 28 May 2025).

Adult patients who underwent lobectomy for pathologically confirmed NSCLC were eligible. Patients undergoing pneumonectomy, segmentectomy, wedge resection, sleeve lobectomy, bilobectomy, or extended resections involving adjacent organs were excluded to ensure a homogeneous study population (*n* = 92). Patients who died within the first 5 postoperative days were excluded because they did not survive beyond the time point required to determine PAL status according to the prespecified definition (*n* = 12).

### 2.2. Data Collection and Study Variables

Demographic, clinical, pulmonary function, pathological, and operative data were extracted from institutional electronic medical records [16,17]. Smoking status was categorized as never, former, or current smoking. Former smokers were defined as patients who had stopped smoking at least 1 year before surgery, whereas patients who were actively smoking or had stopped smoking less than 1 year before surgery were classified as current smokers. Histopathological diagnosis and pathological staging were based on WHO and ninth-edition TNM classifications [18,19].

### 2.3. Surgical Procedure

Lobectomy was performed either through video-assisted thoracoscopic surgery (VATS) or muscle-sparing posterolateral thoracotomy, according to surgeon preference and patient characteristics.

Systematic mediastinal lymph node dissection was routinely performed in accordance with the European Society of Thoracic Surgeons (ESTS) guidelines [20]. Chest drainage was managed with a conventional water-seal system according to the institutional protocol without routine digital drainage or external suction.

### 2.4. Outcome Definition

The primary outcome was prolonged air leak (PAL), defined as an air leak persisting for more than five postoperative days. This definition is consistent with the criteria adopted by both the European Society of Thoracic Surgeons (ESTS) and the Society of Thoracic Surgeons (STS) databases and has become the most widely accepted definition in contemporary thoracic surgery literature [3,4,9,12].

### 2.5. Model Development and Internal Validation

Patients were randomly assigned to the derivation and internal validation cohorts in a 3:1 ratio using a computer-generated random allocation sequence [21]. The derivation cohort was used to develop the SSPAL model and the validation cohort to assess its performance. To complement the split-sample validation, an additional bootstrap-based internal validation analysis was performed using the complete cohort (*n* = 905). For this analysis, a logistic regression model was fitted to the complete cohort using the same five predictors selected during model development in the derivation cohort. The predictor set was kept fixed, but the regression coefficients and intercept were re-estimated using the complete cohort. Subsequently, 1000 bootstrap resamples were generated, and the model was refitted in each resample using the same predictor set. Model discrimination and calibration were assessed in each bootstrap sample and in the original complete cohort to estimate optimism. Optimism-corrected performance estimates were obtained by subtracting the mean bootstrap-estimated optimism from the corresponding apparent performance measures. This supplementary analysis assessed the optimism associated with fitting the model using the fixed predictor set and was distinct from the split-sample validation of the original derivation model. The regression coefficients and intercept of the final SSPAL model, estimated exclusively in the derivation cohort, were applied unchanged to the internal validation cohort. Similarly, the probability threshold determined by maximizing the Youden index in the derivation cohort was applied to the validation cohort without re-estimation or re-optimization.

### 2.6. Statistical Analysis

Statistical analyses were performed using IBM SPSS Statistics version 23.0 (IBM Corp., Armonk, NY, USA) and R statistical software (R Foundation for Statistical Computing, Vienna, Austria; version 4.6.1). R was used for logistic regression modeling, receiver operating characteristic (ROC) curve analyses, bootstrap internal validation, calibration assessment, and comparisons of correlated ROC curves using DeLong’s test. An a priori sample-size calculation was performed using G*Power (version 3.1.9.7; Heinrich Heine University Düsseldorf, Düsseldorf, Germany) based on an anticipated difference in PAL incidence of 10% versus 25%. With a two-sided α level of 0.05 and 80% statistical power, approximately 100 patients per group (approximately 200 patients in total) were required. The available cohort substantially exceeded this minimum sample-size requirement. Continuous variables were assessed for normality using the Kolmogorov–Smirnov test. Continuous variables are presented as mean ± SD or median (IQR), as appropriate. Categorical variables are presented as frequencies and percentages. Comparisons between groups were performed using the independent samples t-test or Mann–Whitney U test for continuous variables, and the Pearson χ^2^ test or Fisher’s exact test for categorical variables, as appropriate. All statistical tests were two-sided, and a *p* value < 0.05 was considered statistically significant.

The assumption of linearity in the logit for continuous candidate predictors (age, BMI, FEV_1_, FVC, smoking pack-years, and tumor size) was assessed by comparing models containing linear terms with models incorporating natural cubic splines with 3 degrees of freedom using likelihood-ratio tests.

Candidate variables were not restricted a priori to preoperative factors, as the initial objective was to identify clinical and perioperative factors associated with PAL. Accordingly, pathological stage, pathological nodal status, and histology were also evaluated in the exploratory univariable analyses. However, none of these postoperative pathological variables were retained in the final multivariable model. All predictors ultimately included in the SSPAL model are available before surgery, allowing the final model to be applied preoperatively. No missing data were identified for the candidate predictors or PAL outcome in the derivation cohort, and no missing data were present for the variables required to calculate SSPAL predictions in the validation cohort. Candidate predictors were initially screened using univariable analyses, and variables with *p* < 0.05 were considered for inclusion in the multivariable logistic regression model. Sex and upper lobectomy were retained in the final model based on their clinical relevance to PAL and the persistence of borderline associations in the multivariable analysis (*p* = 0.070 and *p* = 0.080, respectively). Because smoking status and cumulative smoking exposure (pack-years) represent related dimensions of tobacco exposure, smoking status was retained in the final model as the more clinically straightforward categorical predictor, rather than including both smoking measures simultaneously. No further elimination based solely on multivariable statistical significance was performed. The predictor selection strategy was therefore partly data-driven, although clinical relevance was also considered when determining the final predictor set. During bootstrap internal validation, the final predictor set was kept fixed, and regression coefficients were re-estimated in each of the 1000 bootstrap resamples. The initial univariable screening and predictor selection procedures were not repeated within the bootstrap resamples. The complete logistic regression equation, including the model intercept and explicit coding of all categorical predictors, is presented in the Results section to allow calculation of individual predicted probabilities. Regression coefficients (β) from the final multivariable model were used to construct the SSPAL model and calculate the predicted probability of PAL using the logistic equation. The predicted probability of PAL was then estimated using the standard logistic transformation:

$$P\left(PAL\right)\;=\;\frac{e^{logit}}{1+e^{logit}}$$

Predicted probabilities were expressed as percentages by multiplying the estimated probability by 100.

Model discrimination was evaluated using the area under the receiver operating characteristic curve (AUC). For exploratory risk stratification, the probability threshold was determined in the derivation cohort by maximizing the Youden index (sensitivity + specificity − 1) on the ROC curve. This data-derived threshold was used to describe classification performance and was not prespecified or intended to represent a clinically optimal decision threshold. Calibration was assessed using the Hosmer–Lemeshow goodness-of-fit test, calibration intercept and slope, and calibration plots comparing observed and predicted PAL probabilities across deciles of predicted risk. For the Hosmer–Lemeshow test and the supplementary calibration table, patients in the internal validation cohort were ranked by their predicted probabilities and assigned to ten approximately equal-sized groups using the same grouping procedure. All calibration analyses in the internal validation cohort were performed using predicted probabilities generated directly from the final SSPAL model. In addition to the random split-sample validation, bootstrap internal validation with 1000 resamples was performed using the full cohort to estimate optimism-corrected discrimination and calibration. For comparison with an established PAL risk model, the ESTS PAL risk score was implemented according to its published criteria: 1 point for male sex, 1 point for FEV_1_ <80% of predicted, and 2 points for BMI <18.5 kg/m^2^ (possible total score, 0–4 points) [12]. The original score was developed for patients undergoing video-assisted thoracoscopic lobectomy, whereas the present study included both VATS and open thoracotomy. Accordingly, comparisons in the full cohort should be interpreted in the context of these differences in surgical approach and case mix. Discrimination of the SSPAL and ESTS models was compared using paired DeLong tests for correlated ROC curves in both the derivation and internal validation cohorts. To further address differences in surgical approach between the original ESTS population and the present cohort, a sensitivity analysis restricted to patients undergoing VATS lobectomy was performed. The discrimination of SSPAL and ESTS was assessed using AUCs with 95% confidence intervals, and paired DeLong tests were used to compare the models within the VATS subgroup. The original SSPAL regression coefficients and ESTS scoring criteria were retained without modification.

## 3. Results

### 3.1. Study Population

A total of 1009 patients were assessed for eligibility. Of these, 92 were excluded because they underwent procedures other than standard lobectomy as specified in the exclusion criteria, and 12 patients were excluded because they died within the first 5 postoperative days before PAL status could be determined. The final study cohort therefore comprised 905 patients, who were randomly allocated in a 3:1 ratio to the derivation (*n* = 680) and internal validation (*n* = 225) cohorts (Figure 1). The median age was 64 years (IQR, 58–69), and 81.0% of the patients were male. Overall, prolonged air leak (PAL) occurred in 188 patients (20.8%). The 30-day and 90-day mortality rates were 1.4% and 3.1%, respectively (Table 1).

### 3.2. Derivation and Internal Validation Cohorts

Patients were randomly allocated to the derivation cohort (*n* = 680) and the internal validation cohort (*n* = 225) (Table 2). Baseline characteristics and the incidence of PAL in the derivation and validation cohorts are summarized descriptively in Table 2.

### 3.3. Predictors of PAL and Development of the SSPAL Model

In the univariable analyses, sex, BMI, smoking status, cumulative smoking exposure, operative side, and upper lobectomy were significantly associated with PAL. FEV_1_ and FVC were also evaluated as candidate predictors but were not significantly associated with PAL in the univariable analysis (*p* = 0.328 and *p* = 0.323, respectively; Table 3). No evidence of departure from linearity in the logit was identified for any of the continuous candidate predictors (*p* for nonlinearity: age, 0.438; BMI, 0.293; FEV_1_, 0.803; FVC, 0.316; smoking pack-years, 0.455; and tumor size, 0.891). In a post hoc collinearity assessment including both smoking status and pack-years, no relevant multicollinearity was identified (adjusted GVIF, 1.054 for smoking status and 1.087 for pack-years); when both measures were included simultaneously, pack-years was not independently associated with PAL (*p* = 0.255). Multivariable logistic regression identified lower BMI, current smoking, and right-sided lobectomy as independent predictors of PAL. Specifically, each one-unit increase in BMI was associated with a lower risk of PAL (OR 0.915; 95% CI, 0.871–0.961; *p* < 0.001), whereas current smoking increased the risk approximately fourfold compared with never smoking (OR 4.096, 95% CI 1.359–12.350, *p* = 0.01). Left-sided lobectomy was independently associated with a lower risk of PAL than right-sided procedures (OR 0.637, 95% CI 0.424–0.956, *p* = 0.03). Sex and upper lobectomy demonstrated borderline statistical significance and were therefore retained in the final prediction model (Table 3).

In the derivation cohort, the median predicted probability of PAL was 16.3% (IQR, 10.3–26.6%), and the mean predicted probability was 20.6%, identical to the observed PAL incidence of 20.6%. Patients who developed PAL had a higher median predicted probability than those without PAL (26.4% [IQR, 16.8–45.0%] vs. 15.0% [IQR, 9.5–23.5%], *p* < 0.001).

### 3.4. Model Performance

In the derivation cohort, the model demonstrated acceptable discrimination, with an AUC of 0.723 (95% CI 0.675–0.771) (Figure 2). The ROC-derived probability threshold maximizing the Youden index in the derivation cohort was 16.7%, yielding a sensitivity of 76.4%, specificity of 58.9%, positive predictive value of 32.5%, and negative predictive value of 90.6%. Using this threshold, PAL occurred in 32.5% (107/329) of patients classified as high risk compared with 9.4% (33/351) of those classified as low risk (OR, 4.645; 95% CI, 3.033–7.113; *p* < 0.001).

In direct comparison with the established ESTS PAL risk score [12], SSPAL demonstrated greater discrimination in the derivation cohort (AUC, 0.723; 95% CI, 0.675–0.771) than the ESTS score (AUC, 0.567; 95% CI, 0.521–0.614), with an AUC difference of 0.156 (95% CI, 0.094–0.217; paired DeLong *p* < 0.001). As an additional internal validation analysis, bootstrap validation with 1000 resamples was performed using the full cohort (*n* = 905). The apparent AUC was 0.724, with a mean bootstrap-estimated optimism of 0.007, yielding an optimism-corrected AUC of 0.717. The optimism-corrected calibration slope was 0.957, and the optimism-corrected calibration intercept was −0.001. These optimism-corrected estimates were obtained from the supplementary complete-cohort bootstrap analysis using the fixed predictor set and should be distinguished from the split-sample validation results, which were based on the original derivation model without coefficient re-estimation.

### 3.5. Calibration

Calibration was assessed using graphical comparisons of observed and predicted PAL probabilities. Visual assessment suggested reasonable overall agreement, although deviations were observed across risk deciles, indicating variability in calibration throughout the predicted risk range (Figure 3). The Hosmer–Lemeshow test did not identify statistically significant evidence of model miscalibration (*p* > 0.05); however, a nonsignificant result does not establish adequate calibration, particularly in the presence of variability across predicted risk deciles.

### 3.6. Validation

In the validation cohort, patients who developed PAL had significantly higher median predicted probabilities than those without PAL (24.3% [IQR, 17.2–45.0%] vs. 15.0% [IQR, 9.4–23.6%], *p* < 0.001). When the 16.7% threshold derived from the derivation cohort was applied without re-optimization, PAL occurred in 33.6% (37/110) of patients classified as high risk compared with 9.6% (11/115) of those classified as low risk (OR, 4.792; 95% CI, 2.294–10.009; *p* < 0.001). At this threshold, sensitivity was 77.1%, specificity was 58.8%, positive predictive value was 33.6%, and negative predictive value was 90.4%. The SSPAL model demonstrated an AUC of 0.728 (95% CI, 0.649–0.807) in the internal validation cohort (Figure 4). SSPAL also demonstrated greater discrimination than the ESTS PAL risk score [12] in the internal validation cohort (ESTS AUC, 0.608; 95% CI, 0.532–0.683), with an AUC difference of 0.121 (95% CI, 0.017–0.224; paired DeLong *p* = 0.023). In a sensitivity analysis restricted to patients undergoing VATS lobectomy, 258 patients (44 PAL events) were included from the derivation cohort and 80 patients (15 PAL events) from the internal validation cohort. In the derivation VATS subgroup, SSPAL demonstrated an AUC of 0.696 (95% CI, 0.611–0.781), compared with 0.579 (95% CI, 0.496–0.663) for the ESTS score (ΔAUC = 0.117; paired DeLong *p* = 0.036). In the validation VATS subgroup, the corresponding AUCs were 0.698 (95% CI, 0.552–0.845) and 0.658 (95% CI, 0.536–0.781), respectively (ΔAUC = 0.040; paired DeLong *p* = 0.655). Calibration was satisfactory overall, with a calibration intercept of 0.037 and a calibration slope of 0.967. The Hosmer–Lemeshow test, calculated using the same ten risk groups as Supplementary Table S1, did not indicate statistically significant evidence of miscalibration (χ^2^ = 8.086, df = 8, *p* = 0.425). The mean predicted probability of PAL was 20.8%, compared with an observed PAL incidence of 21.3%. Decile-based assessment showed overall agreement between predicted and observed probabilities, although variability was observed across individual risk deciles (Supplementary Figure S1 and Supplementary Table S1).

## 4. Discussion

This study developed and internally validated a practical prediction model specifically designed to estimate the risk of prolonged air leak (PAL) after lobectomy for non-small-cell lung cancer. The SSPAL model demonstrated moderate discrimination using routinely available preoperative variables. These findings suggest potential value for exploratory preoperative risk estimation; however, its clinical utility remains to be established through external validation and further evaluation of clinical decision thresholds.

Several prediction models have been developed to identify patients at increased risk of prolonged air leak following pulmonary resection [22,23,24,25,26,27]. However, differences in study populations, predictor variables, and intended clinical applications have limited their routine use. Brunelli et al. [22] developed one of the earliest prediction models for PAL and identified advanced age, impaired pulmonary function, low body mass index, pleural adhesions, and upper lobectomy as important risk factors. Subsequently, additional models, including the PALS score and the Epithor model, incorporated broader surgical populations and a greater number of perioperative variables to improve predictive accuracy [23,24,25,26]. Many models rely on intraoperative variables, limiting preoperative risk assessment. In contrast, although candidate predictors in the present study were not restricted a priori to preoperative variables, all predictors retained in the final SSPAL model are routinely available before surgery, enabling individualized preoperative risk assessment and supporting patient counseling and perioperative planning.

Current smoking was an independent predictor of PAL in the present study, increasing the risk more than fourfold. This finding is consistent with previous reports identifying smoking as a major determinant of postoperative air leak following pulmonary resection [9,25]. Smoking contributes to emphysematous destruction, impaired tissue elasticity, and delayed wound healing, increasing the risk of persistent air leak [23,25,28]. These findings further support current recommendations advocating smoking cessation before elective lung resection, not only to reduce pulmonary complications but also to decrease the risk of PAL. However, the wide confidence interval around the estimate for current smoking indicates limited precision, likely reflecting the small number of PAL events among never smokers in the derivation cohort. Accordingly, the magnitude of this association should be interpreted cautiously. Importantly, the current-smoking category included both patients who were actively smoking and those who had stopped smoking less than one year before surgery. Therefore, the observed association with PAL should be interpreted in the context of this operational definition and should not be attributed exclusively to active smoking at the time of surgery.

Lower body mass index was another independent predictor of PAL in our cohort. This observation is in agreement with previous studies demonstrating an inverse relationship between BMI and postoperative air leak [4,12,24,25]. Although the underlying mechanism has not been fully elucidated, patients with lower BMI frequently exhibit reduced pleural fat, diminished soft tissue support, and a higher prevalence of emphysematous changes, all of which may increase the mechanical vulnerability of the residual lung following resection [9,29]. Conversely, Montané et al. have suggested that a modestly higher BMI may exert a protective effect by providing greater pleural apposition and reducing tension across the staple line [30]. Therefore, low BMI should be considered an important preoperative risk factor when estimating an individual’s probability of developing PAL.

In the present study, right-sided lobectomy was independently associated with an increased risk of PAL, whereas upper lobectomy demonstrated borderline statistical significance and was retained in the final prediction model. The association between resection site and PAL has been reported previously, although findings have been inconsistent [4,7,13]. Several investigators have suggested that upper lobectomy, particularly on the right side, predisposes patients to PAL because of greater residual pleural space, incomplete postoperative lung expansion, and increased tension along the staple line [25,31]. Although upper lobectomy did not reach conventional statistical significance in the multivariable analysis (*p* = 0.080), it was retained in the final model because of its established clinical relevance to PAL and the persistence of a borderline association in the multivariable analysis. These findings highlight the importance of anatomical factors in PAL risk assessment.

The SSPAL model demonstrated moderate discrimination in the derivation and split-sample validation cohorts (AUCs of 0.723 and 0.728, respectively). In the validation cohort, the calibration intercept (0.037) and slope (0.967) suggested limited overall systematic miscalibration, although graphical assessment revealed variability across predicted risk deciles. Therefore, calibration performance should be interpreted cautiously, particularly across individual risk strata. These findings suggest that the SSPAL model may provide preliminary preoperative risk estimates; however, its moderate discrimination, limited positive predictive value, and the observed variability in calibration warrant caution regarding clinical interpretation. The model is intended to complement, rather than replace, clinical judgment by providing an objective estimate of PAL risk. Importantly, additional bootstrap validation using the full cohort yielded an optimism-corrected AUC of 0.717 and a calibration slope of 0.957, closely concordant with the performance observed in the split-sample validation cohort. The small estimated optimism in discrimination (0.007) suggests limited overfitting conditional on the selected predictor set, although the bootstrap procedure did not account for uncertainty introduced by the initial predictor selection process. Importantly, the 16.7% cutoff was derived statistically by maximizing the Youden index and should therefore be regarded as an exploratory risk-stratification threshold rather than a clinically established decision threshold. Its clinical utility and the most appropriate action thresholds require evaluation in external prospective cohorts. Furthermore, SSPAL demonstrated greater discrimination than the established ESTS PAL risk score in both the derivation and internal validation cohorts. Importantly, this difference remained statistically significant in the validation cohort, in which SSPAL achieved an AUC of 0.728 compared with 0.608 for the ESTS score (*p* = 0.023). The comparison with the ESTS score should be interpreted cautiously because the original ESTS score was derived in a VATS lobectomy population, whereas 62.7% of patients in our cohort underwent thoracotomy. Differences in surgical approach and case mix may influence model discrimination and limit direct comparability. The higher AUC of SSPAL in this dataset therefore does not establish general superiority over the ESTS score. A VATS-restricted sensitivity analysis and independent external validation are important for assessing comparative performance in more comparable populations. These findings suggest improved discriminatory performance of SSPAL within the present study population; however, comparative performance should be confirmed in independent external cohorts. In the VATS-restricted sensitivity analysis, SSPAL demonstrated higher discrimination than the ESTS score in the derivation subgroup; however, the difference was not statistically significant in the validation subgroup. The smaller AUC difference observed after restricting the analysis to VATS patients highlights the importance of differences in surgical approach and case mix when comparing prediction models. The limited number of PAL events in the validation VATS subgroup further warrants cautious interpretation. These findings do not establish general superiority of SSPAL over the ESTS score, and independent external validation remains necessary.

Because all predictors retained in the final model are available preoperatively, the SSPAL model may be suitable for further investigation as an exploratory preoperative risk-estimation tool. However, given its moderate discrimination and the modest positive predictive value associated with the ROC-derived threshold, the model should not currently be used as an established clinical decision-support instrument. Its clinical utility, appropriate decision thresholds, and potential impact on perioperative management require evaluation in prospective external validation studies.

The major strengths of this study include its large homogeneous cohort, the preoperative availability of all predictors retained in the final model, and successful internal validation, supporting further evaluation of the SSPAL model in external cohorts. Several limitations should also be acknowledged. First, this was a retrospective single-center study, which may limit the generalizability of the findings. Second, excluding 12 patients who died within the first five postoperative days may introduce selection bias, as early death precluded ascertainment of PAL. Consequently, predicted risks apply to patients surviving beyond this period. Third, although the model demonstrated consistent performance across both split-sample and bootstrap internal validation, external validation in independent multicenter cohorts remains necessary before broader clinical implementation. Additionally, potentially relevant variables, such as quantitative emphysema measures, intraoperative air leak grading, staple-line reinforcement, and surgeon-specific factors, were not available or were not systematically recorded and therefore could not be evaluated. Candidate predictor selection was partly based on univariable statistical significance, which may have excluded clinically relevant predictors and introduced instability into the final model. Although sex and upper lobectomy were retained based on clinical considerations, the overall selection strategy remained partly data-driven and may have increased the risk of overfitting. Furthermore, bootstrap validation was performed using the final fixed predictor set rather than repeating the entire variable selection process. Consequently, the optimism-corrected performance estimates may not fully account for the additional uncertainty introduced by predictor selection. An additional limitation concerns the initial sample-size justification, which was based on detecting a difference between two event proportions rather than on methodology specifically developed for multivariable prediction models. Although the derivation cohort included 140 PAL events for six regression parameters in the final model, the resulting event-to-parameter ratio does not fully account for the number of candidate predictor parameters, anticipated model performance, or the potential for overfitting associated with predictor selection. Therefore, this ratio alone cannot establish sample-size adequacy, and residual overfitting and imprecision cannot be excluded. Finally, the study period encompassed several years during which surgical practice and perioperative management may have evolved. Thoracotomy accounted for 62.7% of procedures in the present cohort, reflecting the operative practice at our institution during the study period. As the use of minimally invasive approaches has subsequently expanded, the distribution of surgical approaches in this cohort may not fully reflect contemporary practice. This temporal and practice-related heterogeneity may affect the transportability of the model and further supports the need for external validation in more contemporary cohorts.

In conclusion, the SSPAL model represents a simple candidate risk-prediction model for estimating the preoperative risk of prolonged air leak in patients undergoing lobectomy for NSCLC. The model demonstrated satisfactory discrimination and calibration during internal validation; however, its clinical utility has not yet been established. Prospective multicenter external validation and evaluation of clinically relevant decision thresholds are required before its use in routine clinical practice. The model may facilitate individualized patient counseling and perioperative planning.

## Figures and Tables

**Figure 1:**
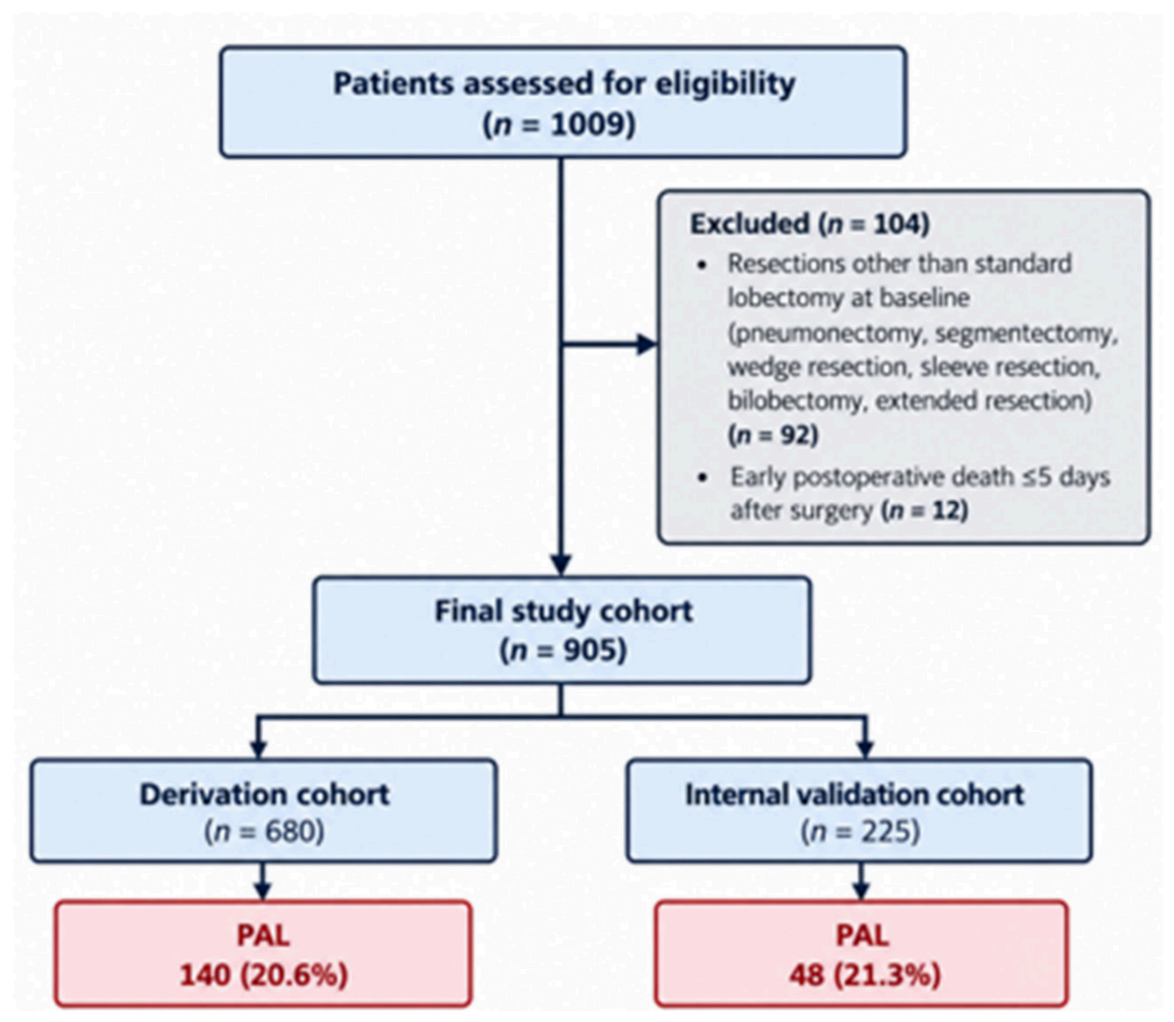
Study flow diagram. Flow diagram showing patient eligibility, exclusions, and random allocation into the derivation (*n* = 680; 140 PAL events) and internal validation (*n* = 225; 48 PAL events) cohorts. Blue and gray boxes indicate patient selection and cohort allocation, whereas red boxed indicate patients who developed prolonged air leak (PAL).

**Figure 2:**
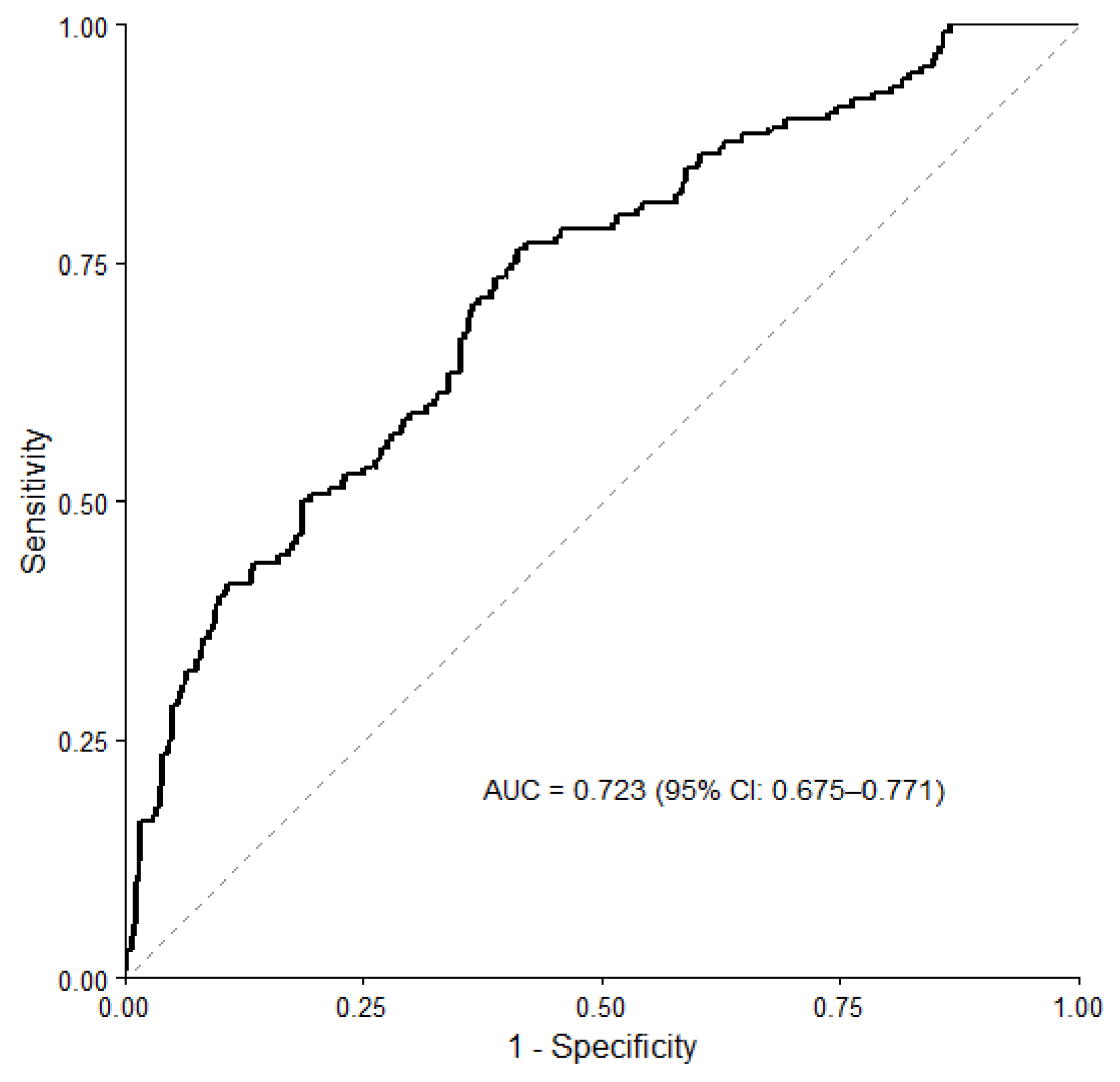
ROC curve of the SSPAL model in the derivation cohort. Receiver operating characteristic curve demonstrating model discrimination in the derivation cohort (*n* = 680; AUC = 0.723; 95% CI, 0.675–0.771).

**Figure 3:**
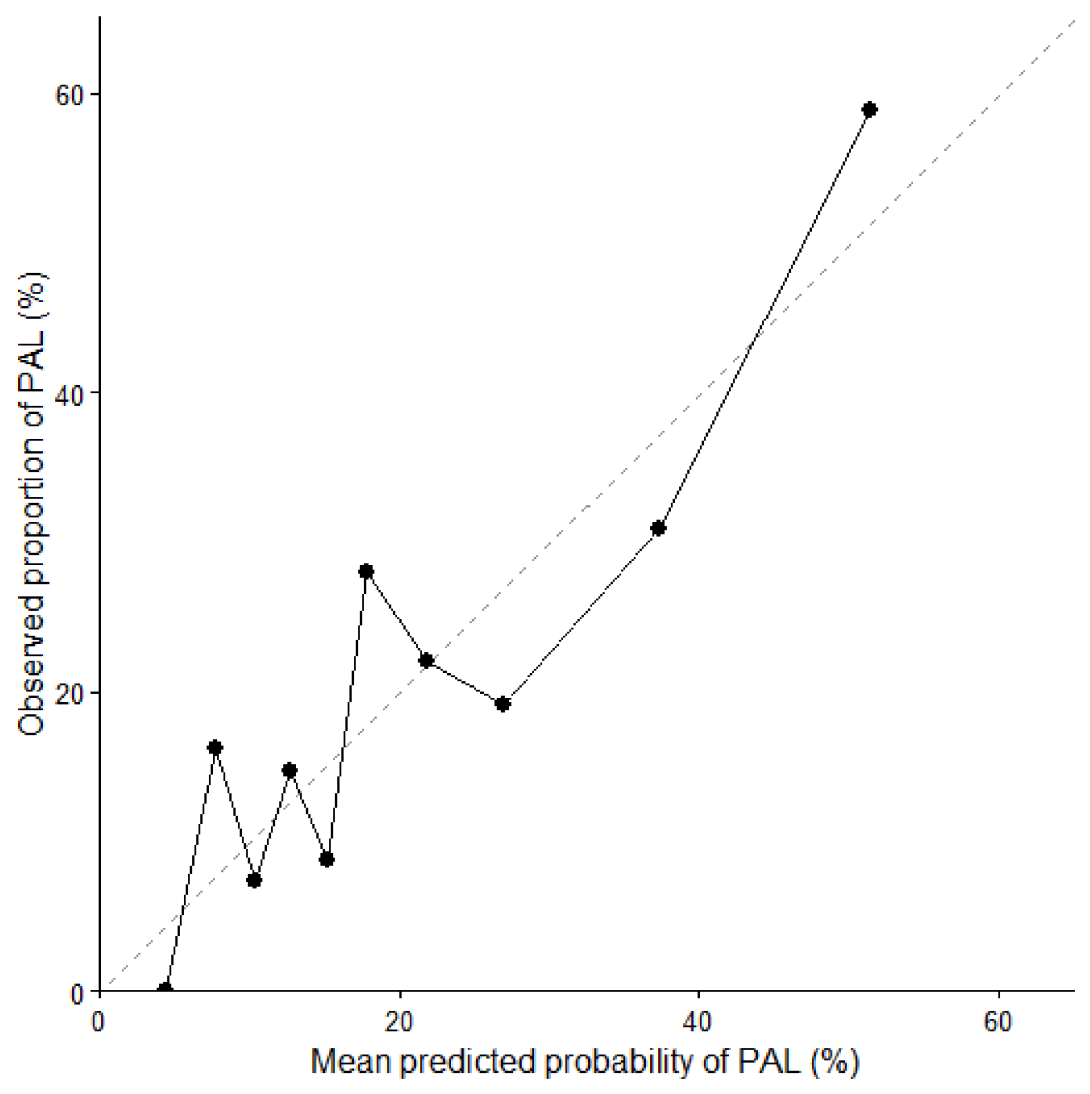
Calibration plot of the SSPAL model in the derivation cohort. Observed PAL proportions are plotted against mean predicted probabilities across deciles of predicted risk in the derivation cohort (*n* = 680). The dashed diagonal represents perfect calibration.

**Figure 4:**
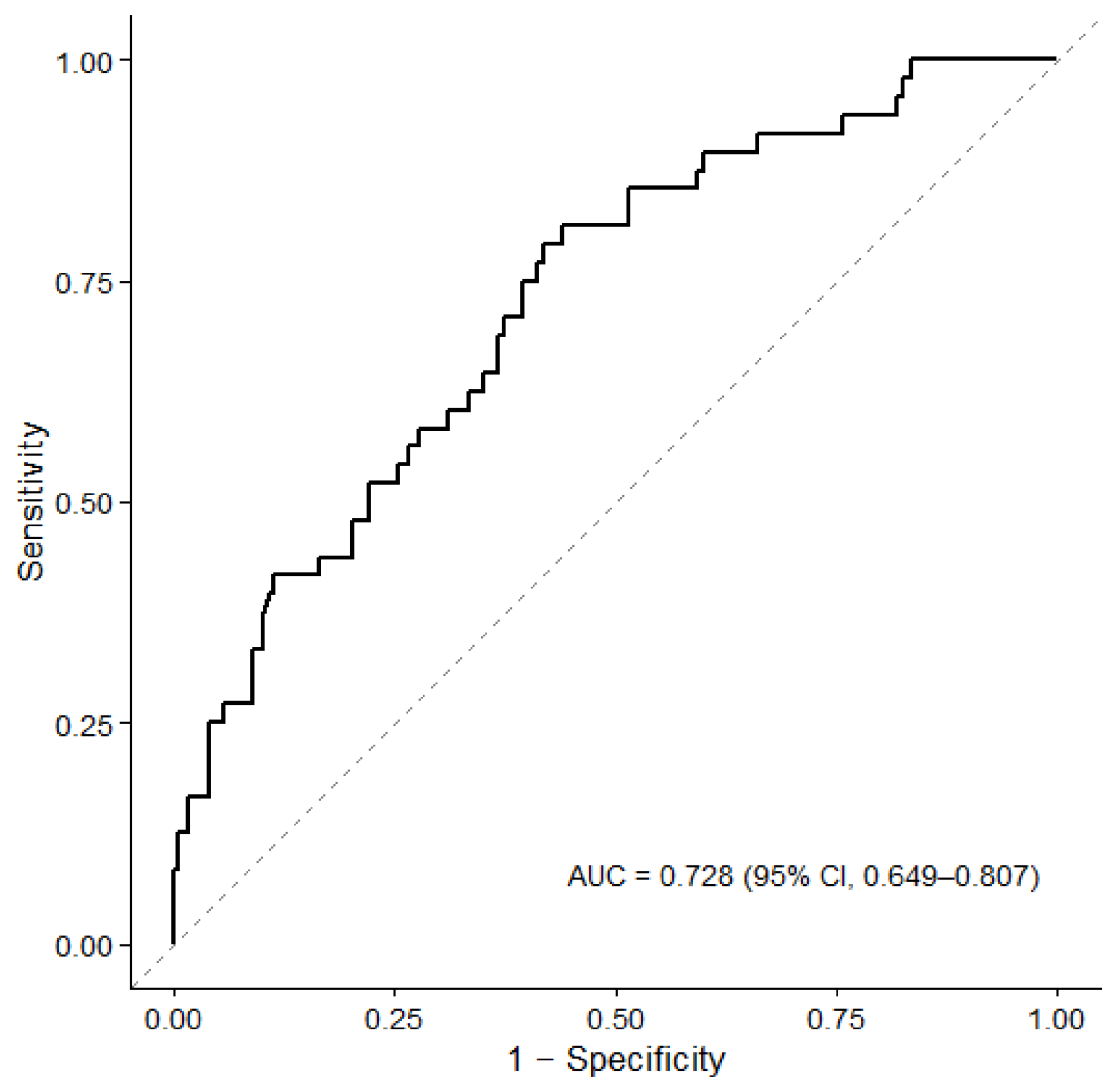
ROC curve of the SSPAL model in the internal validation cohort. Receiver operating characteristic curve demonstrating model discrimination in the internal validation cohort (*n* = 225; AUC = 0.728; 95% CI, 0.649–0.807).

**Table 1:** Baseline demographic, clinical, and pathological characteristics of the study population (*n* = 905).

Variable	Overall Cohort (*n* = 905)
Age, years, median (IQR)	64 (58–69)
Sex, *n* (%)	
Female	172 (19.0)
Male	733 (81.0)
Body mass index, kg/m^2^, median (IQR)	25.9 (23.5–29.4)
FEV_1_, % predicted, median (IQR)	80 (70–91)
FVC, % predicted, median (IQR)	83 (74–92)
Smoking status, *n* (%)	
Never	70 (7.7)
Former	581 (64.2)
Current	254 (28.1)
Smoking exposure, pack-years, median (IQR)	40 (30–59)
Charlson Comorbidity Index, *n* (%)	
0	484 (53.5)
1	320 (35.4)
2	93 (10.3)
3	8 (0.9)
Operative side, *n* (%)	
Right	479 (52.9)
Left	426 (47.1)
Surgical approach, *n* (%)	
VATS	338 (37.3)
Thoracotomy	567 (62.7)
Upper lobectomy, *n* (%)	589 (65.1)
Neoadjuvant therapy, *n* (%)	101 (11.2)
Histology, *n* (%)	
Squamous cell carcinoma	434 (48.0)
Adenocarcinoma	409 (45.2)
Other	62 (6.9)
Tumor size, cm, median (IQR)	3.0 (2.0–5.0)
Pathological N stage, *n* (%)	
N0	690 (76.2)
N1	114 (12.6)
N2	101 (11.2)
Pathological stage, *n* (%)	
I	456 (50.4)
II	214 (23.6)
III	235 (26.0)
Postoperative prolonged air leak, *n* (%)	188 (20.8)
30-day mortality, *n* (%)	13 (1.4)
90-day mortality, *n* (%)	28 (3.1)

Data are presented as median (interquartile range [IQR]) or *n* (%), unless otherwise indicated. BMI, body mass index; CCI, Charlson Comorbidity Index; FEV_1_, forced expiratory volume in 1 s; FVC, forced vital capacity; IQR, interquartile range; VATS, video-assisted thoracoscopic surgery.

**Table 2:** Comparison of baseline demographic, clinical, and pathological characteristics between the derivation and internal validation cohorts.

Variable	Derivation Cohort (*n* = 680)	Internal Validation Cohort (*n* = 225)
Age, years, median (IQR)	64 (58–69)	64 (58–69)
Sex, *n* (%)		
Female	128 (18.8)	44 (19.6)
Male	552 (81.2)	181 (80.4)
Body mass index, kg/m^2^, median (IQR)	25.9 (23.5–29.4)	25.9 (23.6–29.6)
FEV_1_, % predicted, median (IQR)	80 (69.2–91)	80 (70–91.5)
FVC, % predicted, median (IQR)	83 (73–92)	82 (75–93)
Smoking status, *n* (%)		
Never	52 (7.6)	18 (8.0)
Former	439 (64.6)	142 (63.1)
Current	189 (27.8)	65 (28.9)
Smoking exposure, pack-years, median (IQR)	40 (30–50)	40 (32–55)
Charlson Comorbidity Index, *n* (%)		
0	369 (54.3)	115 (51.1)
1	232 (34.1)	88 (39.1)
2	73 (10.7)	20 (8.9)
3	6 (0.9)	2 (0.9)
Operative side, *n* (%)		
Right	362 (53.2)	117 (52.0)
Left	318 (46.8)	108 (48.0)
Surgical approach, *n* (%)		
VATS	258 (37.9)	80 (35.6)
Thoracotomy	422 (62.1)	145 (64.4)
Upper lobectomy, *n* (%)	443 (65.1)	146 (64.9)
Neoadjuvant therapy, *n* (%)	74 (10.9)	27 (12.0)
Histology, *n* (%)		
Squamous cell carcinoma	326 (47.9)	108 (48.0)
Adenocarcinoma	307 (45.1)	102 (45.3)
Other	47 (6.9)	15 (6.7)
Tumor size, cm, median (IQR)	3.0 (2.0–5.0)	3.0 (2.0–4.5)
Pathological N stage, *n* (%)		
N0	518 (76.2)	172 (76.4)
N1	87 (12.8)	27 (12.0)
N2	75 (11.0)	26 (11.6)
Pathological stage, *n* (%)		
I	338 (49.7)	118 (52.4)
II	165 (24.3)	49 (21.8)
III	177 (26.0)	58 (25.8)
Postoperative prolonged air leak, *n* (%)	140 (20.6)	48 (21.3)
30-day mortality, *n* (%)	10 (1.5)	3 (1.3)
90-day mortality, *n* (%)	21 (3.1)	7 (3.1)

**Table 3:** Univariable comparison and multivariable logistic regression analysis of factors associated with prolonged air leak (PAL) in the derivation cohort (*n* = 680).

Variable	No PAL (*n* = 540)	PAL (*n* = 140)	Univariable *p* Value	β Coefficient α	Adjusted OR (95% CI)	Multivariable *p* Value
Age, years, median (IQR)	63 (58–69)	64 (59–69)	0.407		—	—
Sex, *n* (%)			0.003			0.070
Male	426 (78.9)	126 (90.0)			Reference	
Female	114 (21.1)	14 (10.0)		−0.579	0.561 (0.297–1.057)	
Body mass index, kg/m^2^, median (IQR)	26.4 (23.8–30.1)	24.5 (21.7–27.6)	<0.001	−0.089	0.915 (0.871–0.961)	<0.001
FEV_1_, % predicted, median (IQR)	80 (70–92)	79 (68–89.25)	0.328	—	—	—
FVC, % predicted, median (IQR)	82 (73–92)	85 (74.75–92.25)	0.323	—	—	—
Smoking status, *n* (%)			<0.001			
Never	48 (8.9)	4 (2.9)		Reference	Reference	
Former	375 (69.4)	64 (45.7)		0.232	1.261 (0.422–3.773)	0.678
Current	117 (21.7)	72 (51.4)		1.410	4.096 (1.359–12.350)	0.010
Smoking exposure, pack-years, median (IQR)	40 (30–55)	50 (35.7–70)	0.001		—	—
Operative side, *n* (%)			0.010			0.030
Right	274 (50.7)	88 (62.9)		Reference	Reference	
Left	266 (49.3)	52 (37.1)		−0.451	0.637 (0.424–0.956)	
Surgical approach, *n* (%)			0.070		—	—
VATS	214 (39.6)	44 (31.4)				
Thoracotomy	326 (60.4)	96 (68.6)				
Upper lobectomy, *n* (%)	338 (62.6)	105 (75.0)	0.006	0.386	1.471 (0.941–2.297)	0.080
Neoadjuvant therapy, *n* (%)	58 (10.7)	16 (11.4)	0.816		—	—
Histology, *n* (%)			0.133		—	—
Tumor size, cm, median (IQR)	3.0 (2.0–5.0)	3.5 (2.0–5.1)	0.174		—	—
Pathological N stage, *n* (%)			0.361		—	—
Pathological stage, *n* (%)			0.880		—	—

Data are presented as median (interquartile range [IQR]) or *n* (%). Adjusted odds ratios are shown only for variables retained in the final multivariable logistic regression model. BMI, body mass index; CI, confidence interval; FEV_1_, forced expiratory volume in 1 s; FVC, forced vital capacity; OR, odds ratio; PAL, prolonged air leak; VATS, video-assisted thoracoscopic surgery; —, not applicable or not included in the multivariable regression model, as appropriate. α The beta regression coefficients calculated for each variable in the logistic regression analysis were entered individually for each patient. With the sum of the regression coefficients and constant (intercept), the logarithm of the odds (log odds) value was calculated for each patient. The complete SSPAL prediction equation was LP = 0.326 − 0.579 (Female) − 0.089 (BMI) + 0.232 (Former smoker) + 1.410 (Current smoker) − 0.451 (Left-sided lobectomy) + 0.386 (Upper lobectomy), where Female = 1 (male = 0); never smoking was the reference category, with Former smoker = 1 and Current smoker = 1 represented by separate indicator variables; Left-sided lobectomy = 1 (right = 0); and Upper lobectomy = 1 (non-upper lobectomy = 0). The individual predicted probability of PAL was calculated as P(PAL) = 1/[1 + exp (−LP)].

## Data Availability

The data that support the findings of this study are available from the corresponding author upon reasonable request. The data are not publicly available because they contain potentially identifiable clinical information and are subject to institutional and ethical restrictions.

## Supplementary Materials

The following supporting information can be downloaded at: https://www.mdpi.com/article/10.3390/jcm15218367/s1, Figure S1: Calibration plot of the SSPAL prediction model in the internal validation cohort; Table S1: Observed and expected prolonged air leak events across deciles of predicted risk.

## References

[1] World Health Organization Lung Cancer. https://www.who.int/news-room/fact-sheets/detail/lung-cancer.

[2] DQ Adult Treatment Editorial Board (2025). PDQ Non-Small Cell Lung Cancer Treatment.

[3] Zaraca F., Crisci R., Augustin F., Brunelli A., Bertolaccini L. (2023). Prolonged air leak after lung surgery: Prediction, prevention and management. J. Thorac. Dis..

[4] Seder C.W., Basu S., Ramsay T., Rocco G., Blackmon S., Liptay M.J., Gilbert S. (2019). A prolonged air leak score for lung cancer resection: An analysis of The Society of Thoracic Surgeons General Thoracic Surgery Database. Ann. Thorac. Surg..

[5] Brunelli A., Pompili C., Dinesh P., Bassi V., Imperatori A. (2018). Financial validation of the European Society of Thoracic Surgeons risk score predicting prolonged air leak after video-assisted thoracic surgery lobectomy. J. Thorac. Cardiovasc. Surg..

[6] Anegg U., Lindenmann J., Matzi V., Mujkic D., Maier A., Fritz L., Smolle-Jüttner F.M. (2006). AIRFIX: The first digital postoperative chest tube airflowmetry—A novel method to quantify air leakage after lung resection. Eur. J. Cardiothorac. Surg..

[7] Brunelli A., Monteverde M., Borri A., Salati M., Marasco R.D., Fianchini A. (2004). Predictors of prolonged air leak after pulmonary lobectomy. Ann. Thorac. Surg..

[8] Stolz A.J., Schützner J., Lischke R., Simonek J., Pafko P. (2005). Predictors of prolonged air leak following pulmonary lobectomy. Eur. J. Cardiothorac. Surg..

[9] Attaar A., Winger D.G., Luketich J.D., Schuchert M.J., Sarkaria I.S., Christie N.A., Nason K.S. (2017). A clinical prediction model for prolonged air leak after pulmonary resection. J. Thorac. Cardiovasc. Surg..

[10] Amore D., Caterino U., Casazza D., Ievoli R., Imitazione P., Saglia A., Izzo A., Rispoli M., Curcio C. (2023). Persistent alveolar air leak following pulmonary lobectomy: An old problem in a modern era. Monaldi Arch. Chest Dis..

[11] Dezube A.R., Dolan D.P., Mazzola E., Kucukak S., De Leon L.E., Bueno R., Marshall M.B., Jaklitsch M.T., Rochefort M.M. (2022). Risk factors for prolonged air leak and need for intervention following lung resection. Interact. Cardiovasc. Thorac. Surg..

[12] Pompili C., Falcoz P.E., Salati M., Szanto Z., Brunelli A. (2017). A risk score to predict the incidence of prolonged air leak after video-assisted thoracoscopic lobectomy: An analysis from the European Society of Thoracic Surgeons database. J. Thorac. Cardiovasc. Surg..

[13] Gioutsos K., Rieder O., Galanis M., Nguyen T.L., Senbaklavaci Ö., Dorn P. (2025). Risk factors for prolonged air leak after uniportal anatomical segmentectomy. Eur. J. Cardiothorac. Surg..

[14] Orsini B., Baste J.M., Gossot D., Berthet J.P., Assouad J., Dahan M., Bernard A., Thomas P.A. (2015). Index of prolonged air leak score validation in case of video-assisted thoracoscopic surgery anatomical lung resection: Results of a nationwide study based on the French national thoracic database, EPITHOR. Eur. J. Cardiothorac. Surg..

[15] Muriana P., Rossetti F., Viscardi S., Novellis P., Veronesi G. (2023). Prolonged air leak after robotic lung resection: A narrative review. J. Thorac. Dis..

[16] Graham B.L., Steenbruggen I., Miller M.R., Barjaktarevic I.Z., Cooper B.G., Hall G.L., Hallstrand T.S., Kaminsky D.A., McCarthy K., McCormack M.C. (2019). Standardization of spirometry 2019 update: An official American Thoracic Society and European Respiratory Society technical statement. Am. J. Respir. Crit. Care Med..

[17] Charlson M.E., Pompei P., Ales K.L., MacKenzie C.R. (1987). A new method of classifying prognostic comorbidity in longitudinal studies: Development and validation. J. Chronic Dis..

[18] WHO Classification of Tumours Editorial Board (2021). Thoracic Tumours.

[19] Detterbeck F.C., Woodard G.A., Bader A.S., Dacic S., Grant M.J., Park H.S., Tanoue L.T. (2024). The proposed ninth edition TNM classification of lung cancer. Chest.

[20] Lardinois D., De Leyn P., Van Schil P., Porta R., Waller D., Passlick B., Zielinski M., Junker K., Rendina E., Ris H. (2006). ESTS guidelines for intraoperative lymph node staging in non-small cell lung cancer. Eur. J. Cardiothorac. Surg..

[21] Urbaniak G.C., Plous S. (2013). Research Randomizer, *version 4.0*.

[22] Brunelli A., Varela G., Refai M., Jimenez M.F., Pompili C., Sabbatini A., Aranda J.L. (2010). A scoring system to predict the risk of prolonged air leak after lobectomy. Ann. Thorac. Surg..

[23] Rivera C., Bernard A., Falcoz P.E., Thomas P., Schmidt A., Bénard S., Vicaut E., Dahan M. (2011). Characterization and prediction of prolonged air leak after pulmonary resection: A nationwide study setting up the index of prolonged air leak. Ann. Thorac. Surg..

[24] Varela G., Jiménez M.F., Novoa N., Aranda J.L. (2005). Estimating hospital costs attributable to prolonged air leak in pulmonary lobectomy. Eur. J. Cardiothorac. Surg..

[25] Zheng Q., Ge L., Zhou J., Zhang Y., Lyu M., Chen C., Wang T., Liu L. (2022). Risk factors for prolonged air leak after pulmonary surgery: A systematic review and meta-analysis. Asian J. Surg..

[26] Shindo Y., Fumimoto S., Hanaoka N., Toyohara K., Sato K., Katsumata T. (2025). Which risk score best predicts prolonged air leak after lobectomy? A comparative evaluation study. Eur. J. Cardiothorac. Surg..

[27] Ponholzer F., Ng C., Maier H., Lucciarini P., Öfner D., Augustin F. (2023). Risk factors, complications and costs of prolonged air leak after video-assisted thoracoscopic surgery for primary lung cancer. J. Thorac. Dis..

[28] Yoshida T., Tuder R.M. (2007). Pathobiology of cigarette smoke-induced chronic obstructive pulmonary disease. Physiol. Rev..

[29] Parini S., Azzolina D., Massera F., Mastromarino M.G., Papalia E., Baietto G., Curcio C., Crisci R., Rena O., Alloisio M. (2023). The overweight paradox: Impact of body mass index on patients undergoing VATS lobectomy or segmentectomy. Semin. Thorac. Cardiovasc. Surg..

[30] Montané B., Toosi K., Velez-Cubian F.O., Echavarria M.F., Thau M.R., Patel R.A., Rodriguez K., Moodie C.C., Garrett J.R., Fontaine J.P. (2017). Effect of obesity on perioperative outcomes after robotic-assisted pulmonary lobectomy. Surg. Innov..

[31] Drewbrook C., Das S., Mousadoust D., Nasir B., Yee J., McGuire A. (2016). Incidence risk and independent predictors of prolonged air leak in 269 consecutive pulmonary resection patients over nine months: A single-center retrospective cohort study. Open J. Thorac. Surg..

