# Supplementary material for: A Preoperative Risk-Prediction Model (SSPAL) for Prolonged Air Leak After Lobectomy for Lung Cancer: Development and Internal Validation
Source: J Clin Med. 2026 Oct 27;15(21):8367. doi: 10.3390/jcm15218367 (PMC13764232; doi:10.3390/jcm15218367)
Supplement: Supplementary file 1 [file jcm-15-08367-s001.zip › jcm-4595539-supplementary.pdf]

Supplementary Table S1: Observed and expected prolonged air leak events across deciles of predicted risk.

Decile	n	Observed PAL, n	Expected PAL, n	Observed PAL, %	Mean predicted risk, %
1	23	0	0.97	0.0	4.2
2	23	3	1.72	13.0	7.5
3	23	2	2.36	8.7	10.3
4	23	2	2.96	8.7	12.9
5	23	4	3.58	17.4	15.5
6	22	8	4.02	36.4	18.3
7	22	4	4.86	18.2	22.1
8	22	5	6.38	22.7	29.0
9	22	8	8.52	36.4	38.7
10	22	12	11.40	54.5	51.9

Expected PAL events were calculated as the sum of individual predicted probabilities within each decile. All predicted probabilities were generated directly from the final SSPAL model.

Supplementary Figure S1. Calibration plot of the SSPAL prediction model in the internal validation cohort

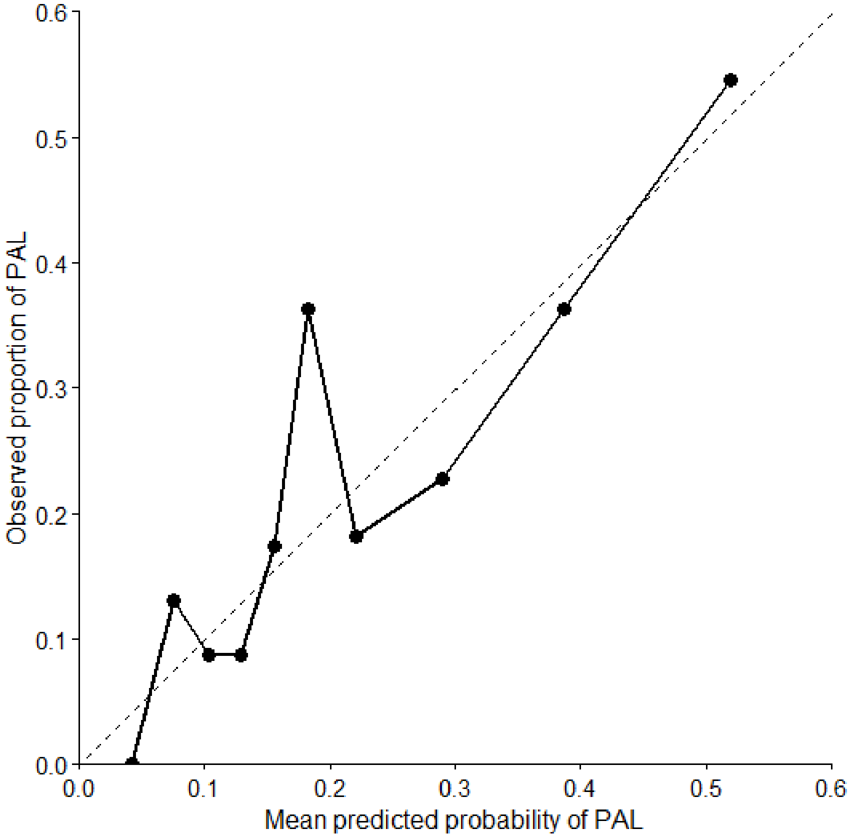

Patients were ranked according to their individual predicted PAL probabilities and divided into ten approximately equal-sized groups. Expected PAL events were calculated as the sum of individual predicted probabilities within each group. Percentages were calculated using unrounded values.
